# Oral Health-Related Quality of Life and Oral Health Indices in Patients With Opioid Use Disorder Undergoing Methadone Maintenance Treatment

**DOI:** 10.1155/ijod/5339532

**Published:** 2025-09-27

**Authors:** Hodis Ehsani, Mohammad Mehdi Dindarloo, Anahita Lotfizadeh, Faezeh Alizadeh, Abbas Alipour, Kamaledin Alaedini, Maede Salehi, Ali Jafari, Alireza Arezumandi, Tahereh Molania

**Affiliations:** ^1^Department of Periodontics, Dental Research Center, Mazandaran University of Medical Sciences, Sari, Iran; ^2^Faculty of Dentistry, Mazandaran University of Medical Sciences, Sari, Iran; ^3^Department of Biostatistics, School of Paramedical Sciences, Shahid Beheshti University of Medical Sciences, Tehran, Iran; ^4^Dentist, Sari, Iran; ^5^Community Medicine Department, Medical Faculty, Mazandaran University of Medical Sciences, Sari, Iran; ^6^Addiction Research Institute, Mazandaran University of Medical Sciences, Sari, Iran; ^7^Department of Oral Medicine, Dental Research Center, Mazandaran University of Medical Sciences, Sari, Iran

**Keywords:** opioid, opioid use disorder, oral health, quality of life

## Abstract

**Background:** Addiction to opioids causes several adverse effects, one of the most important of which is oral side effects. However, oral health has a noticeable impact on the quality of life. The purpose of the present study is to investigate the indicators of oral health and oral health-related quality of life (OHRQoL) among patients with opioid use disorder to help treatment planning.

**Methods:** A total of 425 people suffering opioid use disorder were selected from addiction treatment centers in Sari. After recording demographic data, the OHRQoL questionnaire (OHIP[oral health impact profile]-14) was administered as an interview. Then nine questions related to xerostomia were asked and were followed by an oral examination to measure indices, including SBI (sulcus bleeding index), GI (gingival index), PI (plaque index), and DMFT (decayed, missed, and filled teeth), and an unstimulated salivary flow rate (USFR) sample was collected for weight measurement to determine patients' hyposalivation. Variables were described using mean, standard deviation, and frequency. Kolmogorov–Smirnov, independent *t*, and Pearson's correlation coefficient tests were used in this study. Multivariate linear regression was used to evaluate the factors related to the quality of life score of opioid users. Statistical analysis was performed by SPSS16 with a significance level of 0.05.

**Results:** The correlation between age, ADD-OHIP, and USFR with xerostomia was statistically significant (*p* < 0.05). GI, SBI, and DMFT indices in patients with hyposalivation (determined by measuring USFR) were lower than those without hyposalivation (determined by measuring USFR) and the differences were not statistically significant (*p* > 0.05). The results of the linear regression model showed that gender, xerostomia, and DMFT are the main determinants of OHIP.

**Conclusion:** Gender, xerostomia, and DMFT are the main determinants of OHIP. Oral health and compliance with oral hygiene in people with opioid use disorder is poor and affects their quality of life. Therefore, providing them proper access to oral health services is necessary.

## 1. Background

Addiction is a destructive and progressive chronic complication related to the uncontrollable need to use drugs and the inability to reduce the amount of use; this condition is physical and psychological dependence on a psychoactive substance [1]. Drug addiction is associated with ecosocial issues and a variety of medical side effects. The most common illegal addictive substances are cannabis and opioids [2].

Opioids are natural or synthetic narcotics with analgesic and nervous system depressant properties that have the potential to create euphoria [3]. Opioid addicts experience severe neurological symptoms known as opioid withdrawal syndrome in case of deprivation; these symptoms include mydriasis, lacrimation, and nasal secretions, increased blood pressure, pulse, and ventilation. Thus, they use methadone, which is considered a type of opioid, to control these symptoms when quitting addiction to previous opioids. Methadone maintenance treatment (MMT) in managing opioid dependence is an alternative treatment that requires daily administration of methadone under the close supervision of a therapist. Methadone treatment allows the patient to reduce drug use and stop the occurrence of dangerous drug-related behaviors [4]. In addition, opioids cause a variety of direct side effects, including physical and psychological problems. These include cardiac diseases, respiratory depression, liver problems, nephropathy, infectious diseases due to weak immune system conditions, such as hepatitis, AIDS (acquired immunodeficiency syndrome), and tuberculosis, depression, loss of appetite, loss of pain sensation, and oral health problems [5, 6]. The primary oral side-effect of opioids can be caused by the direct impacts of these drugs on the oral cavity; primary complications, including bruxism and xerostomia, cause secondary complications, such as multiple caries and periodontal problems. However, the role of the poor oral hygiene and high carbohydrate diet of addicts is significant in this case [2, 6, 7]. Drug addicts usually do not seek treatment even if symptoms, such as swelling and pain occur in the mouth [8]. Furthermore, people with opioid use disorders mainly use alcohol and cigarettes along with drugs; the harmful outcomes of alcohol and smoking on oral health have been proven, so they aggravate the oral side effects of opioids [5, 6, 9]. A recent study by Maboudi et al. indicates the harmful effect of poor oral hygiene and the correlation of *P. gingivalis*, the bacteria which plays significant role in periodontal disease development, with malignant conditions, such as esophageal squamous cell carcinoma development [10].

Dental diseases are one of the most important diseases that occur along with the use of drugs, and it is a reason to improve the link between oral and dental treatments, and addiction treatment plan [11].

Quality of life is a multidimensional category that includes social, psychological, physical, and environmental dimensions of an individual's life. Also, poor oral health can affect a person's psychological and functional health [1, 9]. The definition of quality of life concentrates on the effect of an illness on a person's daily activities, with particular attention to mental and physical health. The overall well-being of a person and satisfaction with life are also considered in this definition [12]. Considering the chronic and recurring nature of addiction, and its impact on different dimensions of a person's life, it is clear that the quality of life should be addressed in studies related to the addiction. Many previous studies examined health-related quality of life, especially in patients with opioid use disorder [13–17].

Oral health may impact people's performance, mental and social status by causing pain and discomfort. Oral health has a considerable effect on the quality of life. Among previous studies, a couple of studies have demonstrated the relationship between oral health-related quality of life (OHRQoL) and treatment planning for people suffering from drug addiction [1]. To develop a plan to improve oral health and quality of life successfully, the initial status of oral health indicators and quality of life of drug addicts should be evaluated [18].

In this study, the incidence of oral diseases, caries, and periodontal conditions in people suffering from opioid use disorder who are undergoing MMT was examined. Furthermore, hyposalivation was determined in the present study by exclusively measuring unstimulated salivary flow rate (USFR). The reason for preferring to measure the USFR over the stimulated salivary flow rate is that, while stimulated saliva is helpful for studying the functional reserve of salivary glands, unstimulated saliva production is a reliable way to assess the condition of the salivary glands. Additionally, hyposalivation symptoms are more closely correlated with USFRs than with stimulated salivary flow rates [19, 20].

Given the direct effect of these facors, it is essential to help improve the quality of life and foresee the necessary accommodations to prevent the occurrence and development of oral diseases.

## 2. Methods

This study is a descriptive cross-sectional study evaluating people suffering from opioid use disorder living in Sari and its suburbs. They had referred to addiction treatment centers in different regions of the city in years 2021–2022 and were undergoing treatment. The inclusion criteria of this study were to provide a recent history of using opioids for at least 1 year and no history of using other drugs, such as hashish, methamphetamine, and alcohol [1, 21]. People with cardiovascular diseases, chronic kidney disease, or other systemic diseases that require patients to take medicines, especially diabetic patients and those taking antianxiety medicines, antidepressants and antihistamines, and people with complete edentulism, were excluded from this study. Furthermore, this study was conducted according to our previous study [22].

The aim of the study and its steps were clarified for all participants at the beginning, followed by obtaining their written consent. The patients were assured that their information would remain confidential.

The results of the study by Sharma et al. were used to determine the sample size [1]. A sample size of 384 people was calculated by estimating the quality of life dependent on optimal oral health to be 50%, the accuracy set to 0.05, and a confidence interval of 95%. Nevertheless, considering the possibility of noncooperation and the providence of incomplete information, the sample size was increased by 10%, rounding to a total of 425 participants.

Sampling was done to homogenize the participants using the stratified method. The classes of the studied population were considered according to their residential area (urban, suburban, and rural classes). While taking into account that out of 102 MMT centers in Sari, 77 (75%) are located in urban areas, 10 (10%) are located in rural areas, and 15 centers (15%) are located in suburban areas (proportional stratified sampling). Accordingly, 17 centers were selected, and from each, 25 patients entered the study. The required data were collected by interviews, questionnaires, and oral clinical examination performed by a senior dental student trained by a periodontist.

Demographic information (including age and sex), as well as information, such as marital status, number of children, employment status, education level, hygiene (tools used such as a toothbrush or dental floss and their frequency of use), history of smoking and details of drug use (the type of substance used, the duration of drug use, etc.) were recorded [1, 6, 23].

In order to evaluate the OHRQoL, the OHIP-14 questionnaire was used, and the validity and reliability of the Persian version were confirmed. This questionnaire includes seven subgroups of functional limitation, physical pain, mental discomfort, physical disability, mental disability, social disability, and handicap, and each subgroup contains two questions [24].

Two methods were utilized to analyze the answers. The additive (ADD) method, in which the answers were scored as never = 0, rarely = 1, sometimes = 2, often = 3, and most of the time = 4. For this method, the sum of the OHIP-14 score was between 0 and 56, and participants scoring closer to zero had better quality of life. In the other evaluation method called SC (simple count), a score of zero was assigned to the answers of never and rarely, and a score of one was assigned to the answers of sometimes, often, and most of the time. This method was used to simplify the answers of the question asked in the questionnaire for the participants. The OHIP-14 score for this method ranged from 0 to 14, and similarly, a lower score indicated a higher quality of life in patients [25].

Then, the patients were examined clinically, and DMFT (decay, missed, and filled teeth) index, PI (plaque index), GI (gingival index), and SBI (sulcus bleeding index) were measured on Ramfjord teeth (teeth number 3, 9, 12, 19, 25, 28), and in the absence of one teeth, an adjacent tooth was considered [26]. The criterion of tooth decay was the clinically visible deficiency (cavity or unsupported enamel), and plaque removal was not performed before the examination [6].

The tools used for the clinical examination included a disposable mirror, an explorer, and a WHO (world health organization) standard periodontal probe from Falcon brand. During the clinical examination, the amount of unstimulated saliva secretion and xerostomia of patients were measured. To identify patients with xerostomia, nine questions were asked by the dental student, and the patients who answered positive to five out of nine questions were diagnosed with xerostomia [27]. The questionnaire used in the present study was taken from a study conducted by So et al. [27]. The questions about symptoms and behaviors of xerostomia are presented in Table 1 (Table 1).

The USFR to determine hyposalivation, should be administered first thing in the morning following an overnight fast; it should be conducted without consuming food or liquids for at least 1 h prior to the test; it should also be conducted without smoking for at least 1 h prior to the test [20]. The saliva was collected while patients were sitting in a resting position, and they were asked to reduce the mouth movement and do not swallow or suck. The dentist would then proceed to collect saliva from their mouth for 60 s and then pour it into a pre-weighed tube. The patients were asked to spit and repeat this for 5 min continuously. The saliva sample was kept in the freezer at −70°C to be measured later. The amount of USFR was checked using the gravimetric method. When the unstimulated whole saliva flow rate in a typical person surpasses 0.1 mL/min, hyposalivation is indicated by a resting secretion rate of less than 0.1mL in a minute [20, 28].

Data were entered into SPSS16 statistical software. Kolmogorov–Smirnov test was used to assess the normality of quantitative variables. Variables were described using mean, standard deviation and frequency. Independent *t*-test was used to compare quantitative variables between two groups. In addition, Pearson's correlation coefficient was used to check the correlation between quality of life and quantitative variables including PI, GI, DMFT, SBI, age, USFR, Xerostomia, and OHIP. Finally, multivariate linear regression was used to evaluate the factors related to the quality of life score in opioid users. A significance level of less than 0.05 was considered.

## 3. Results

Of the 425 patients, 399 (93.9%) were men, and 26 (6.1%) were women. The average age of the participants was 42.42 ± 11.3 years (18–82 years). The duration of drug use was 1–32 years, with an average of 7.52 ± 11.5. All the studied patients were under treatment with methadone, and 351 subjects (82.6%) also used cigarettes at the same time. The average frequency of using a toothbrush was 4.22 ± 2.97 times per week, and the average frequency of using dental floss was 2.24 ± 0.63 times per week. 48 patients (11.3%) were using partial prostheses. The results of evaluating quantitative variables are shown in Table 2.

*t*-Test was used to investigate the relationship of xerostomia and hyposalivation (determined by measuring USFR) with the quantitative variables; the results are shown in Tables 3 and 4, respectively. The average age of patients with xerostomia was 45.48 ± 11.64 years. Age, ADD-OHIP, and USFR,unstimulated hyposalivation status indicator, had a significant correlation with xerostomia (*p* < 0.05) (Table 3).

The average age of patients with hyposalivation was 48.81 ± 13.16, and the correlation between age and USFR index, unstimulated hyposalivation status indicator was not significant (*p*=0.214). PI and ADD-OHIP index and xerostomia were higher in patients with hyposalivation; only xerostomia had a significant correlation with hyposalivation (*p*=0.003). GI, SBI, and DMFT indices in patients with hyposalivation were lower than those without hyposalivation and the differences were not statistically significant (Table 4).

To investigate the relationship between the quality of life and the quantitative variables, Pearson's correlation test was used. The correlation between age, xerostomia, and USFR with OHIP was not statistically significant, but the correlation between PI, GI, SBI, and DMFT with quality of life was statistically significant (*p* < 0.05) (Table 5).

By evaluating the factors related to OHIP and using multivariate linear regression, the effect of age, sex, DMFT, PI, GI, SBI, OHIP-SC, and xerostomia was modified. According to the results of multivariate linear regression, the quality of life in women was 3.209 units higher than in men. Women had a lower quality of life than men. For each unit of age increase, 0.007 unit of quality of life score decreased. Finally, the OHIP score increased by 0.286 in patients with xerostomia. In other words, patients with xerostomia had worse quality of life than patients without xerostomia. This difference was statistically significant (*p*-Value = 0.0250). OHIP score increased by 0.252 units with each unit of DMFT, and this difference was statistically significant (*p*-Value = 0.0054). OHIP score increased by 1.934 times with increasing GI score, 0.454 times with increasing PI score, and 2.647 times with increasing smoking score, none of which were statistically significant. Also, the quality of life in patients who used partial prostheses was 1.064 units lower than those who didn't. The quality of life in patients with hyposalivation was 1.064 units higher than those without hyposalivation. In general, the results of the linear regression model showed that gender, xerostomia and DMFT are the main determinants of OHIP. It should be noted that the variables in the multivariate linear regression model constitute 25.4% of the alterations related to the OHRQoL and dental health. (Table 6).

## 4. Discussion

The results of various studies have shown the significant effect of opioid consumption on oral health indicators and OHRQoL. Opioid counsumption causes oral issues that are associated with OHRQoL. According to the findings of the present study, the average score of OHIP in patients was 21.43 ± 11.57. To evaluate the factors related to the quality of life, the effect of variables such as age, sex, DMFT, PI, GI, SBI, xerostomia, and USFR were modified by multivariate linear regression analysis. In general, the results of the linear regression analysis showed that the variables of gender, xerostomia, and DMFT are the main determinants of OHIP. According to the findings, women had a poorer quality of life than men. Also, patients with xerostomia and with a higher DMFT index had a poorer quality of life.

Numerous issues affecting oral and dental health are brought on by drug use [29], including tooth loss, widespread tooth decay, oral mucosal infections, candidiasis, mucosal dysplasia, xerostomia, erogenous, bruxism, tooth wear, and temporomandibular disorders [30]. Drug users generally have poor dental health, and their gums are coated in a white slough that when removed, reveals erythema and inflammation [1].

The periodontal health of patients who use opioids is put at risk because addictive medications, especially opioids, harm cell division, cause tissue destruction, hinder its repair and regeneration [31]. Additionally, narcotic medications activate alpha-adrenergic receptors in salivary gland vessels, which results in vasoconstriction and decreases USFR (hyposalivation), significantly weakening protective functions like neutralizing acids produced by bacterial plaque and remineralization of tooth enamel thus, increasing the risk of caries [1, 7]. In the current investigation, it was found that methadone, a medication used in addiction treatment centers, increases caries in patients by decreasing salivary acidity, immunological function, and immunoglobulins (IgG, IgM, and IgA) [4]. In addition, factors such as improper nutrition, lifestyle, more tendency to consume sugar, negligence in hygiene and lack of access and visit to the dentist affect the oral health of individuals [6, 30].

In the present study, 82.6% of patients were smokers. According to a study conducted by Nigar et al. [32], comparing the USFR of active and passive smokers, it was demonstrated that the number of cigarettes was negatively correlated with saliva production. Changes in saliva characteristics leads to oral health issues including dental and periodontal problems; the results of a study designed by Lazureanu et al. [33], evaluating USFR in patients with periodontal diseases, showed that the severity of the periodontal disease was correlated with lower salivary flow rate (0.28 mL/min).

Since opioid drugs have considerable impacts on periodontium, teeth, and saliva function, these factors were assessed in the current investigation.

The participants in this study were 42.42 ± 11.13 years old on average. This study, like other investigations in China and India, investigated a wide age range. Studies have revealed that individuals with a more extended history of drug use and younger onset of usage had worse oral hygiene practices and more pronounced consequences on their oral and dental health [7, 18]. According to the research findings, older patients who began using drugs earlier in life and for more extended periods had higher DMFT scores [4, 6, 7, 11, 34]. The average level of xerostomia was also higher in older participants in the current investigation. Medicines used to treat systemic conditions like hypertension, diabetes, and depression may also contribute to xerostomia by influencing the salivary glands, causing a decrease in salivary flow, and causing xerostomia [35, 36]. Patients with xerostomia also report dysarthria, dysphagia, halitosis, and lack of denture retention, in addition to the fact that xerostomia is a risk factor for dental caries and affects the DMFT. As a result, previous studies and the current study have found that xerostomia and the subsequent rise in DMFT significantly affect the quality of life [30, 35, 36].

The results of the current study indicate that gender is yet another variable that influences OHIP in such a way that women experience lower quality of life. According to Nordrehaug et al., the women who use opioids have lower quality of life [37]. Compared to males, women are more conscious of their appearance. Since the mouth and teeth are visible body parts that greatly contribute to a person's appearance and self-confidence, any disorder or disease in these areas prevents the patient from showing them when smiling, makes them emotionally sensitive, restrains them from participating in community activities, and ultimately lowers their quality of life [30]. The rate of tooth decay and tooth loss, as well as the presence of decayed and retained roots, have all been reported to be higher in women than men, even though the results of the studies show that oral and dental hygiene is more considered in women [4, 11, 18]. This discrepancy between the worse health of the teeth and better hygiene may be attributed to physiological causes, such as increased dental caries in women after pregnancy and xerostomia brought on by menopause, followed by increased decay and DMFT [38, 39]. In general, drug users in this study, regardless of gender, had a great mean DMFT, similar to those in previous investigations [6, 30]. Although this finding was significantly higher than those of the studies done in China and India [7, 31].

The constituents of opium affect the oral microbiota and disturb the microbial ecology of the mouth. Smoking and opium consumption are associated with periodontal diseases caused by *P. gingivalis* [40]. In the present study and previous studies, the periodontal condition of drug users has been carefully examined. In the present study, the mean PI, GI, and SBI were 0.91 ± 1.68, 0.790 ± 1.292, and 1.349 ± 1.16, respectively.

Gingival bleeding was noted by Ye et al. [7] in 97.53% of patients, by Ma et al. [4] in 99% of patients, and by Gupta et al. [31] in 42% of drug users. On the other hand, the results of this investigation revealed an average SBI of 1.349 ± 1.16, which signifies the moderate bleeding (“0–3” represents normal to severe inflammation). Most patients (82.6%) in the current study also smoked. The normal gingival inflammatory response and subsequent gingival bleeding in smokers are concealed by the constriction of gingival capillaries and disruption of gingival bleeding caused by smoking [41].

Smoking's damaging effects on the gums may reduce patients' GI and SBI to the lower levels than predicted.

The presence of shallow dental pockets with a higher prevalence and deep dental pockets with a lower prevalence in drug users have been mentioned in different studies [4, 6, 7, 31]. Also, in a study conducted by Shekarchizadeh et al. [6] on opioid users in Iran, the results showed that periodontal diseases were higher in both healthy people and drug users compared to other studies conducted in other countries. Periodontal problems, followed by tooth mobility and tooth loss, affect the DMFT of the patients and, as mentioned earlier, the quality of life.

Although, factors like low socioeconomic status, lack of access to a dentist, people's chaotic lifestyles, fear of dental procedures, and the attitudes of dental professionals and their concern about the spread of infectious diseases are among the factors that prevent patients from visiting the dentist, the results of this study and previous studies show the necessity of frequent visits to the dentist. Oral and dental disorders can be caused by a variety of factors, including a lack of awareness of the importance of oral and dental health and poor oral hygiene practices.

In light of the issues raised in this study, it is hoped that dental services will soon be offered in addiction treatment centers and that both drug users and dental service providers will undergo the necessary cultural change regarding the importance of oral health and its impact on quality of life of drug users.

Due to the lack of sampling facilities, this study was conducted in addiction treatment centers without advanced dental equipment, which increases the possibility of errors in examinations. In addition, due to the lack of information related to oral and dental problems among people suffering from addiction and the descriptive-cross-sectional nature of this study, it is not possible to fully interpret the data. The sample was restricted to opioid users undergoing MMT, and cannot be generalized to other groups, such as prisoners or users of other substances. Individual variations in the time of previous drug use, the duration of methadone treatment, and the time of detoxification and rehabilitation may have impacted the results.

## 5. Conclusion

In this study, poor DMFT and periodontal problems among people with opioid use disorder can be related to a lack of hygiene, long history of drug use, and wrong lifestyle factors. Also, the indicators of oral health, hygiene, and the history of opioid use disorder affects the OHRQoL of these patients. Gender, xerostomia, and DMFT are the main determinants of OHIP. Treatment of periodontal diseases and dental decays in patients with opioid use disorder can be done by establishing a link between addiction treatment programs, oral and dental health services, and effective health policies to prevent complications and improve medical services, and it seems that use of dental treatments for drug addicts can advance their quality of life and self-esteem.

## Figures and Tables

**Table 1:** Questionnaire used to assess xerostomia in the present study.

Symptoms of xerostomia
Does your mouth feel dry when eating a meal?
Do you have difficulties swallowing any foods?
Does the amount of saliva in your mouth seem to be reduced most of time?
Does your mouth feel dry at night or on awakening?
Does your mouth feel dry during the daytime?
Do you usually wake up thirsty at night?

**Behaviors related to xerostomia**

Do you need to sip liquids to aid in swallowing dry foods?
Do you chew gum or use candy to relieve xerostomia?
Do you keep a glass of water by your bed?

**Table 2:** The results of evaluating quantitative variables.

Variables	Mean	SD	Median
Age	42.42	11.13	41.00
PI	1.68	0.91	1.70
GI	1.29	0.79	1.20
SBI	1.16	1.35	1.00
DMFT	16.72	7.13	17.00
ADD-OHIP	21.43	11.57	20.00
SC-OHIP	6.96	3.57	7.00
USFR	1.096066	0.734528	0.999500
Xerostomia	3.16	2.38	3.00

**Table 3:** The results of the *t*-test used to investigate the relationship between xerostomia with the quantitative variables.

Variables	Patients with xerostomia		Patients without xerostomia		*p*-Value
	Mean ± SD	Number	Mean ± SD	Number	
Age	45.48 ± 11.64	124	41.16 ± 10.68	301	<0.001
PI	1.78 ± 0.74	124	1.63 ± 0.97	301	0.124
GI	1.39 ± 0.75	124	1.25 ± 0.80	301	0.114
SBI	1.31 ± 1.40	124	1.10 ± 1.32	301	0.151
ADD-OHIP	23.02 ± 12.77	124	20.77 ± 10.99	301	0.039*⁣*^*∗*^
SC-OHIP	3.70 ± 7.24	124	3.52 ± 6.84	301	0.293
DMFT	17.51 ± 7.03	124	16.39 ± 7.16	301	0.141
USFR	0.58 ± 0.81	124	60.7 ± 1.21	301	<0.001

*⁣* ^*∗*^ *p*-Value < 0.05.

**Table 4:** The results of the *t*-test used to investigate the relationship between hyposalivation with the quantitative variables.

Variables	Patients with hyposalivation		Patients without hyposalivation		*p*-Value
	Mean ± SD	Number	Mean ± SD	Number	
Age	48.81 ± 13.16	16	42.28 ± 11.05	408	0.214
PI	1.98 ± 0.74	16	1.66 ± 0.91	408	0.163
GI	1.17 ± 0.67	16	1.29 ± 0.79	408	0.552
SBI	0.63 ± 0.95	16	1.18 ± 1.35	408	0.107
ADD-OHIP	23.75 ± 14.96	16	21.32 ± 11.43	408	0.410
SC-OHIP	3.47 ± 7.69	16	3.58 ± 6.92	408	0.401
DMFT	18.31 ± 8.18	16	16.63 ± 7.08	408	0.356
Xerostomia	2.12 ± 4.88	16	2.36 ± 3.09	408	0.003*⁣*^*∗*^

*⁣* ^*∗*^ *p*-Value < 0.05.

**Table 5:** Investigation of the relationship between the quality of life and the quantitative variables by Pearson's correlation test.

Quantitative variables	*r* (Pearson correlation coefficient)	*p*-Value
Age	0.063	0.196
PI	0.158*⁣*^*∗*^	0.001
GI	0.176*⁣*^*∗*^	<0.001
SBI	0.108*⁣*^*∗*^	0.026
DMFT	0.187*⁣*^*∗*^	<0.001
USFR	−0.057	0.251
Xerostomia	0.074	0.094
OHIP-SC	0.851	<0.001

*⁣* ^*∗*^ *p*-Value < 0.05.

**Table 6:** The results of the linear regression analysis.

Variables	Beta	*p*-Value
Age	−0.007	0.899
Sex	3.209*⁣*^*∗*^	0.016
Smoke	2.647	0.075
DMFT	0.252*⁣*^*∗*^	0.005
PI	0.454	0.497
GI	1.934	0.077
SBI	−0.323	0.597
Xerostomia	0.286*⁣*^*∗*^	0.025
USFR	−1.379	0.541
Prosthesis	−1.064	0.568

*⁣* ^*∗*^Signifies the *p*-value <0.05.

## Data Availability

All data generated or analyzed during this study are included in this published article.

## Nomenclature

OHIP: Oral health impact profile
SBI: Sulcus bleeding index
GI: Gingival index
PI: Plaque index
DMFT: Decayed, missed, and filled teeth
USFR: Unstimulated salivary flow rate
OHRQoL: Oral health-related quality of life
MMT: Methadone maintenance treatment
AIDS: Acquired immunodeficiency syndrome
ADD: Additive
SC: Simple count
WHO: World Health Organization
Ig: Immunoglobulin.
